# Molecular mechanisms of cystic fibrosis – how mutations lead to misfunction and guide therapy

**DOI:** 10.1042/BSR20212006

**Published:** 2022-07-01

**Authors:** Carlos M. Farinha, Isabelle Callebaut

**Affiliations:** 1 BioISI – Instituto de Biosistemas e Ciências Integrativas, Faculdade de Ciências, Universidade de Lisboa, 1749-016 Lisboa, Portugal; 2 Sorbonne Université, Muséum National d’Histoire Naturelle, UMR CNRS 7590, Institut de Minéralogie, de Physique des Matériaux et de Cosmochimie, IMPMC, 75005 Paris, France

**Keywords:** CFTR, Cystic Fibrosis, Molecular basis of disease, Mutations, Theratypes

## Abstract

Cystic fibrosis, the most common autosomal recessive disorder in Caucasians, is caused by mutations in the cystic fibrosis transmembrane conductance regulator (*CFTR*) gene, which encodes a cAMP-activated chloride and bicarbonate channel that regulates ion and water transport in secretory epithelia. Although all mutations lead to the lack or reduction in channel function, the mechanisms through which this occurs are diverse – ranging from lack of full-length mRNA, reduced mRNA levels, impaired folding and trafficking, targeting to degradation, decreased gating or conductance, and reduced protein levels to decreased half-life at the plasma membrane. Here, we review the different molecular mechanisms that cause cystic fibrosis and detail how these differences identify theratypes that can inform the use of directed therapies aiming at correcting the basic defect. In summary, we travel through CFTR life cycle from the gene to function, identifying what can go wrong and what can be targeted in terms of the different types of therapeutic approaches.

## CF: the disease

Cystic fibrosis (CF) is the most common autosomal recessive disorder among Caucasians, affecting about 80,000 individuals worldwide. The frequency of this disorder is highly variable and is often a function of ethnic and geographic origin of the affected patients [1].

CF is a monogenic disorder caused by mutations in the cystic fibrosis transmembrane conductance regulator (*CFTR*) gene, which encodes the CFTR protein, a chloride and bicarbonate channel responsible for regulation of ion transport across the apical membrane at the surface of certain epithelia [2,3]. The most common disease-causing mutation is F508del – the deletion of three nucleotides that leads to the deletion of the phenylalanine residue at position 508 in the polypeptidic chain – responsible for about two-thirds of all CF chromosomes [4].

CF is a multi-organ disorder affecting exocrine epithelia and characterized by a diversity of symptoms, as all epithelial tissues are affected by loss of CFTR function – which can be attributed not only to the loss of channel function but also to the direct or indirect impact of CFTR in several cellular processes. Thus, the clinical manifestations can be caused by the loss of CFTR itself or to the absence of the chloride (and bicarbonate) conductance that it mediates [5].

Clinically, the most devastating CF phenotype is the impairment of lung function and airways disease. Mutations in the CFTR gene lead to defective CFTR expression or function. This triggers abnormalities in ion transport and thus in water absorption which results in the dehydration of the airway surface liquid (ASL) altering mucus composition and impairing protection against bacterial infections. The increased viscosity of the mucus layer results in small airways obstruction and facilitates colonization by pathogens, especially *Pseudomonas aeruginosa*. Response to infections triggers inflammatory response, initiating a vicious cycle that culminates in extended bronchiectasis and severe respiratory failure [6].

The decrease or loss of CFTR expression and function – caused by CFTR mutations, as described below for different mutation types – has a major impact on epithelial salt and fluid transport. Normal airway epithelia have the ability to regulate water content in the ASL, mediated by Na^+^ absorption through the epithelial Na channel (ENaC), that exits the cell via the basolateral sodium potassium pump, with Cl^−^ following passively via tight junctions. Cl^−^ is secreted from the cell by the apical membrane channels like CFTR or calcium-activated Cl^−^ channels (CaCC, such as TMEM16A/anoctamin 1), with Cl^−^ entering the cell principally via the basolateral Na^+^-K^+^-2Cl^−^ cotransporter. So, a balance between ENaC, CFTR and other channels is crucial to control the ASL volume. Acting as a regulator of other channels (including ENaC [7,8] and also CaCCs), CFTR is a central player in the regulatory mechanisms for electrolyte and water movement in leaky secretory epithelia.

CF is also characterized by pancreatic insufficiency (PI) – probably due to the important role that CFTR also plays in bicarbonate secretion – elevated levels of chloride in the sweat – where the lack of functional CFTR blocks NaCl reabsorption (which are coupled in this tight epithelium, differing from the airways) – meconium ileus, low body mass index (BMI), infertility in men mostly because of bilateral absence of *vas deferens* (CBAVD), and undescended testicles or hydrocele, amenorrhea in women and severe nutritional involvement [3,9,10]. However, there is a significant variability of CF symptoms among different individuals, which probably reflect the multitude of functions of CFTR – and how different mutations have distinct impact in the expression or function of the protein.

## *CFTR*: the gene

The *CFTR* gene was cloned in 1989 using Chromosome walking and jumping and complementary DNA hybridization [11]. The gene spans a region of around 189 kb in the long arm of chromosome 7 (position 7q31.2) and is formed by 27 exons. The coding sequence is 4,443 nucleotides long and encodes a protein with 1,480 aminoacids.

Initial studies focusing on CFTR expression in epithelial cell lines revealed that the gene is constitutively transcribed at low levels and highlighted several characteristics of the promoter, including the lack of a TATA element, a high G+C content, the presence of multiple start sites for transcription and of several sites for the Sp1 transcription factor. These observations suggested that the promoter is similar to that of an ‘housekeeping’ gene and that although the gene being expressed at low levels in organs that are associated with the CF phenotype, its expression can be modulated through transcription [12].

Analysis of CFTR expression patterns in highly expressing cells identified multiple sites of transcription initiation between positions -95 and +50 of the CFTR gene, and the presence of a splicing-related alternative transcript. Expression levels of the *CFTR* gene are apparently correlated with the chromatin structure and the methylation status of the promoter – implying a role for *CFTR*’s physical context in the regulation of its transcription [13].

Since the *CFTR* gene exhibits a complex pattern of expression – with both temporal and spatial regulation and likely a complex function of several overlapping regulatory pathways, several studies aimed at identifying potential regulatory elements. Mapping of DNase-I-hypersensitive sites (DHSs) identified several of these elements flanking the gene, including DHSs at -79.5 and -20.9 kb with respect to the CFTR translational start site and a regulatory element in the first intron of the gene at 185+10 kb [14–16] and further DHSs lying 3′ to the CFTR gene at 4574+5.4, +6.8, +7.0, +7.4 and +15.6 kb. Some of these DHSs exhibit a certain degree of tissue specificity [17,18].

**Figure 1:**
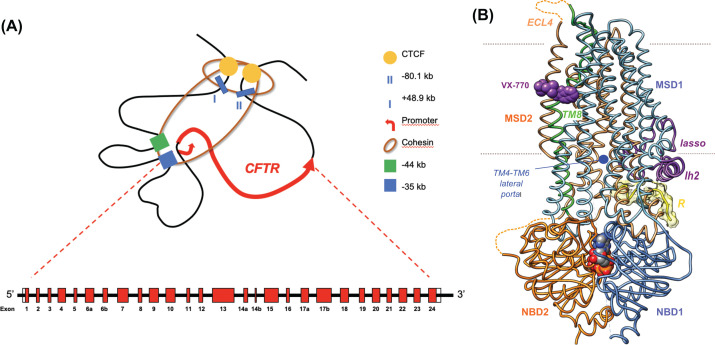
CFTR gene and protein (**A**) 3D organization of the *CFTR* gene – the so-called topologically associated domain – in airway cells, showing the promoter, the boundaries (I and II) and architectural proteins CTCF and cohesin (adapted from [23]). (**B**) Ribbon representation of the human CFTR 3D structure (ATP-bound, phosphorylated form) in complex with VX-770 (PDB: 6O2P [124]), illustrating striking features of the channel, as discussed in the text. The two ATP molecules are shown at the interface between NBD1 and NBD2. Transmembrane (TM) helices extend in the cytoplasm, forming long intracellular loops (ICLs) which contact the NBDs through coupling helices. ECL4 stands for extracellular loop 4. Fragment of the R domain was modeled as a poly-alanine into a density of the cryo-EM map (yellow). This figure (and Figure 2) was prepared using Chimera https://www.rbvi.ucsf.edu/chimera.

The existence of several DHSs located far apart from the promoter, in intronic and in flanking intergenic regions, soon revealed to be crucial to intricate 3D structure of the CFTR locus [19] forming a complex looped structure in cells that express the gene but is absent from cells where the gene is inactive [20]. The architectural proteins CTCF (CCCTC binding factor) and cohesin contribute to establish a topological domain (TAD) that regulates chromatin dynamics to remodel nucleosomes, recruit cell-selective transcription factors and activate intronic enhancers [21,22]. The 3D organization of the *CFTR* gene and regulatory elements together with architectural proteins (Figure 1A) is tissue specific and has a major role in regulating *CFTR* expression (reviewed in [23]).

## CFTR: the protein

The CFTR (ABCC7) protein is a member of the ABC transporter superfamily, which evolved toward the specific function of a phosphorylation-activated and ATP-gated ion channel [24]. Accordingly, as supported first by theoretical studies [25–30] and afterwards demonstrated by experimental 3D structures [31–35], CFTR shares many features of ABC transporters but exhibits specific characteristics (Figure 1B). Among the common features is the domain organization with two nucleotide-binding domains (NBDs) and two membrane-spanning domains (MSDs), which have arisen from an ancestral duplication and resulted in a pseudo dimer architecture. CFTR, however, possesses a unique, long regulatory (R) domain linking the two halves of the protein, which has characteristics of intrinsically disordered sequences and displays multiple PKA phosphorylation sites [36,37]. At least two critical steps of CFTR activation lead to pore opening : (i) phosphorylation of the R domain [38], relieving inhibitory interactions with the rest of the protein and allowing its displacement from a position wedged between the NBDs [31,34,36,39], (ii) ATP-binding to NBDs, promoting a stable dimer formed by the NBDs [40,41] and the two pairs of MSD intracellular loops (ICLs) [42,43]. The PKA dependence of channel activity of CFTR constructs lacking NBD2 is in favor of an additional phosphorylation-dependent regulation mechanism independent of NBD dimerization and suggests that the R domain also interacts with MSD ICLs after ATP-dependent dimerization [44,45]. Contrasting with the alternating-access mechanism of ABC transporters, with gates at both ends which are alternatively opened and closed, the intracellular gate of CFTR is degenerated [46,47], revealing lateral portals (or entrances) at the level of the MSDs ICLs, which connect the cytoplasm to a large inner vestibule [30,34]. This vestibule, including basic residues which were proposed to act as anion binding sites [48] or create favorable electropositive potential [49], is at the base of a central cavity formed by transmembrane helices and lined by residues associated with channel selectivity [46,50]. Experimental 3D structures of the ATP-bound, but still closed human and zebrafish CFTR [33,34], supported by functional evidence [51,52], have shown that only one of two portals, the one formed between transmembrane helices 4 (TM4) and 6 (TM6), is open to the cytoplasm, while the other one, in the vicinity of the C-terminal, occluding end of the R domain, is not. At the extracellular end of the pore, the narrow region, considered as the selectivity filter, is made of residues from TM1, TM6 and TM12 but also TM8, as revealed by the cryo-EM 3D structures of human and zebrafish CFTR [33,34,53]. TM8 is unwound over approximately 10 amino acids, with R933 establishing a salt bridge with TM7 E873, and occupies, together with TM7, a non-canonical position with respect to the general topology of the ABC superfamily type IV exporters (after the new classification proposed by Thomas and colleagues [54]). TM8 was proposed to play a role in the gating of the pore, especially as its upper part rotates of approximately 55° between the ATP-free and ATP-bound conformations [34]. However, the exact mechanisms allowing a full conductance remains to be understood. Long MD simulations of the zebrafish CFTR 3D structures in a lipidic environment indicated stability of this conformation and suggest that transition to an open state may be influenced by lipids [55]. Metadynamics simulations have identified open conformations of the channel, involving the two portals and possible exits towards the extracellular milieu, especially involving the participation of lipid tails [56]. Also of note are 3D structures from thermostabilized, dephosphorylated and phosphorylated chicken CFTR, in presence of ATP, showing a fully helical TM8 in an ABC usual position, while TM7 is nearly orthogonal to the lipid bilayer [35]. This suggests that the fully conductive state might adopt a conformation not yet observed at an atomic level. Additional 3D structures and simulations are thus needed to further understand the CFTR gating cycle and associated conformational transitions.

An additional original feature of CFTR, which is also found in other members of the ABC-C family such as MRP1, SUR1 and SUR2, is a significant divergence in the ATP-binding site 1 motif, formed by the NBD1 Walker A, Walker B and H-loop and by the NBD2 ABC signature and D-loop [24]. These non-canonical features are responsible for increased affinity for ATP and slowed ATP hydrolysis at this site [57,58], which is also covered by large regulatory insertion (RI), bearing a phosphorylation site. RI is involved in profound conformational transitions in NBD1, not only providing functional gain [59] but also increasing vulnerability to misfolding [60]. A last particular feature of CFTR, also common to other members of the ABC-C family, is a sequence, called ‘lasso’, N-terminal to the first membrane-spanning domain (MSD1), which is partially embedded in the membrane bilayer. The first part of the lasso, including lasso helix 1 (Lh1), forms a circular ‘noose’ structure, interacting with transmembrane helices TM2 and TM6 from MSD1 and TM10 and TM11 from MSD2, as well as with the R domain, as observed in the ATP-bound 3D structure [34]. The C-terminal part of the lasso, including Lh2, is tucked under the MSD1 elbow helix. Several experimental data, in particular considering amino acid variants, have indicated a critical role of this N-terminal lasso in the CFTR folding, gating and stability [61–66]. The lasso is likely to adopt multiple conformation during folding and functioning, allowing regulation of CFTR by cytoplasmic partners (reviewed and commented in [61,67]).

## Molecular mechanisms of disease – how do CFTR mutations cause CF?

CF is caused by mutations in the *CFTR* gene. To date, more than 2100 variants have been described in the gene, most of which are presumed to cause disease.

Although all are predicted to impair chloride and bicarbonate, the mechanism through which they cause disease is diverse. To facilitate the understanding of the molecular details leading from mutation to misfunction, mutations have been grouped into functional classes – highlighting the molecular defects – that evolved into theratypes [68] – highlighting the therapeutic strategy to be used (but not necessarily the therapeutic agent).

**Scheme 1:**
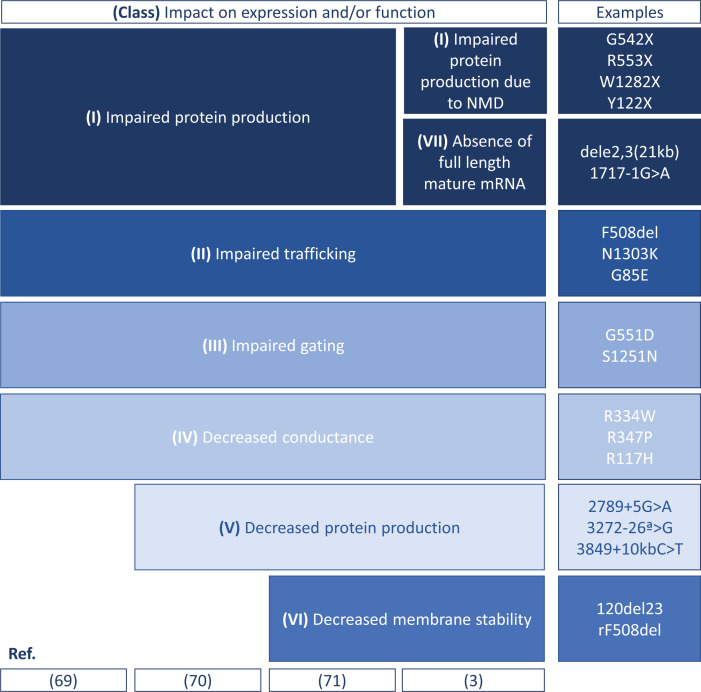
Classes of mutations in the *CFTR* gene, according to the molecular and cellular defect caused

Scheme 1 summarizes the evolution of the grouping into classes, from the original 4 classes to the most recent 7 classes.

The following sections are devoted to the description of the mechanisms through which mutations lead to disease and how the understanding of such mechanisms has been used to design therapeutic approaches targeted at the basic defect. Structural data help to understand the molecular basis of CFTR dysfunction when the protein is mutated and the mechanisms of action of modulators. On a fundamental point of view, mutations and modulators are also relevant tools for understanding the molecular details underlying CFTR folding and gating. This global knowledge can be used to improve modulator efficiency and design novel CFTR modulators, in particular for mutations which are not responsive to current modulators. As many mutations lead to multiple defects [72], a comprehensive knowledge of the possible binding sites and mechanisms of action of modulators, either alone or combined, is of outmost importance to develop mutant-specific combinatorial therapies.

### Mutations affecting folding/trafficking

The most common mutation F508del causes disease by interfering with the folding of CFTR, thus preventing its trafficking and leading to premature degradation.

As CFTR emerges from the ribosome, it is co-translationally inserted into the ER membrane and folded. The fidelity of the process is assessed by both cytosolic and ER chaperones that allow the wild-type (wt) protein to progress through the secretory pathway and tag the mutant one for degradation [73]. Unfolding is mainly caused as mutations disrupt crucial intramolecular interactions that are needed for intra- and inter-domain assembly of a complex protein such as CFTR. The delay in the folding process exposes hydrophobic regions for too long causing alterations in protein–protein interactions that go beyond association with specific chaperones such as Hsp70 [74] or Hsp90 [75] and leads to global changes in CFTR interactome [76]. This distinction between folded and unfolded CFTR also involves a role for ER lectins such as calnexin, which is dependent on CFTR glycan moieties. Failure to exit the ER is directly linked to the inability of CFTR to properly fold resulting in the continued exposure of retention motifs that prevent the packaging into COPII vesicles at the ER exit sites.

The recognition and disposal of misfolded CFTR are performed by a set of at least four checkpoints that are part of the ER quality control (ERQC) system:

– The first checkpoint involves interaction with molecular chaperones, especially those belonging to the Hsp70 family. F508del-CFTR association with Hsp70 is prolonged and most of the mutant protein is targeted to degradation at this early step which involves the replacement of pro-folding cochaperones such as Hdj-1/2 [77,78] by pro-degradation ones such as Bag-1 or CHIP. This set of machinery forms what has been described as a ‘chaperone trap’ [79,80].
– The second checkpoint depends on the interaction of CFTR glycoconjugates with calnexin and a significant proportion of wt-CFTR is also sent to degradation at this step [81,82].
– A third checkpoint involves recognition of ER retention motifs such as the arginine framed tripeptides (AFTs). When CFTR folding occurs without disturbance, these motifs are buried within the protein that is then allowed to leave the ER. When misfolding occurs, exposed AFTs are recognized and mediate retention to prevent CFTR exit from the ER. Previous studies have identified proteins involved in such recognition step [83,84].
– Finally, CFTR is packed into COPII vesicles, a process that is mediated by the recognition of a diacidic export code located in NBD1. Abrogation of this motif leads to partial or total retention in the ER, not due to misfolding but to the inability to be recognized by the coat proteins [83,85,86].

Besides F508del, many mutations are thought to lead to CFTR misfolding, although the position of the mutations may cause differences in the intramolecular interactions that are disrupted and thus on the interacting proteins that are mediators in the specific retention processes, contributing to the notion that not all trafficking mutations are ‘created equal’ [87].

The first attempts of using low molecular weight compounds to repair CFTR were targeted at folding/trafficking mutations and aimed at correcting the folding process either acting directly at CFTR – pharmacological chaperones – or modulating the cellular milieu – either chemical chaperones or, more recently, proteostasis modulators. Correctors were identified and optimized from such initial attempts.

**Figure 2:**
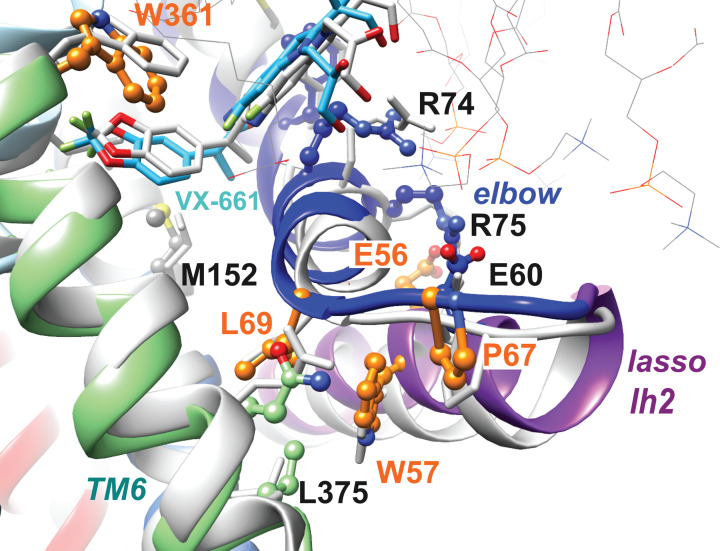
Rare mutations affecting the CFTR N-terminal part and rescued by VX-661 Are shown in orange the positions of five amino acids, associated with rare class II mutations, on the 3D structure of human CFTR in complex with VX-661 (colored: experimental 3D structure after blind docking of VX-661 and MD simulation in presence of lipids (tiny sticks) [89], light gray: experimental 3D structure of the protein–drug complex (PDB: 7SV7, [92]). W57 is sandwiched between P67 and L69, in tight contact with the TM6 C-terminal end, thereby forming a lock between the N- and C-terminal ends of MSD1. E56 forms a salt-bridge with R75, itself interacting with E60. These bonds participate in the large network linking MSD1 to the lasso lh2, ICL1 and ICL4 [89].

Mutations affecting folding and processing (grouped in Class II) are particularly abundant at the ICLs:NBDs interfaces [88]. They are also present in the N-terminal part of CFTR, including the lasso (especially, Lh2) and MSD1, highlighting the intrinsic fragility of this region (reviewed in [89,90], Figure 2). Moreover, the importance of this N-terminal region in the co-translational and cooperative folding process, in particular through ICL1 and ICL4, makes it a critical actor for the stability of the whole CFTR protein [61,89,91]. Recently, two independent studies have identified the binding site of the correctors lumacaftor (VX-809) and related tezacaftor (VX-661) in a groove formed at the basis of MSD1 by the elbow helix, TM1, TM3 and TM6 [89,92] (Figure 2). While one of the two studies used cryo-electron microscopy [92], the other one was based on molecular docking and molecular dynamics simulations and provided additional information on the participation of lipids in the binding site and on the allosteric pathway coupling drug binding in MSD1 to NBD1, where F508del lies [89]. Correctors such as VX-809 and VX-661 intervene on the MSDs:NBD1 assembly that is affected by the F508del mutation, in particular on the interface that NBD1 establishes with the intracellular loop ICL4 of MSD2. ICL4 is tightly associated with ICL1 (with a salt-bridge between TM2 K162 and TM11 E1075), and ICL1 is tightly associated with the lasso Lh2 (with a salt-bridge between TM2 K162 and Lh2 D47), itself interacting with the MSD1 elbow helix [89]. In fact, the F508del mutation has a general effect on multiple interfaces [93,94]: not only the ICL4:NBD1 interface is affected but also the NBD1:NBD2 interface [95], explaining why mutations at the ICL4:NBD1 interface also perturb gating and require potentiator to be rescued. While VX-809/VX-661 have an effect on domain interfaces, they have however no effect on the NBD1 thermal unstability [96,97], and efficient correction of the F508del defects thus require additional pharmacological chaperones targeting NBD2-specific and NBD1-specific defects [83,98,99]. The newly developed corrector elexacaftor (VX-445), following a NBD1-specific mechanism and included in the combination Trikafta [100], provides additive rescue of the F508del defect, but has also co-potentiator activity additive to that of ivacaftor (VX-770) [101–103]. Molecular docking followed by molecular dynamics simulations have identified a potential binding site of VX-445 in MSD1 (distinct from the MSD1 VX-809/VX-661 binding site) but also in NBD1 [89]. This last site, which corresponds to the region that is targeted by the dual potentiator-corrector CFFT-01 [104], was also predicted to accommodate VX-809/VX-661 and is consistent with experimental data gained for both types of correctors [100,105]. Other potential binding sites are likely to exist for other correctors. For instance, MCG1516A, a corrector additive to VX-661, was proposed to target the interface between NBD1 and NBD2 principally as it is not additive to the G550E revertant, in contrast to the R1070W one (at the interface between ICL4 and NBD1) [106]. Other corrector binding sites have been proposed within NBD1 [107] and at the NBD1:ICL4 interface [108–110].

Some mutations may impair CFTR trafficking at a later stage, decreasing its residency time at the plasma membrane (PM). Such mutations do not impair trafficking from the ER to the PM, through the Golgi, but affect critical interactions that anchor CFTR at the PM.

CFTR membrane levels are a balance between anterograde trafficking, endocytosis, and recycling, each of which rely of specific protein interactions [73]. Among these, PDZ domain containing proteins are particularly relevant. These interact with CFTR’s C-terminus which contains a PDZ-binding motif and ultimately leads to the anchoring of the protein to the cytoskeleton and mediate its interaction with other channels and transporters [111]. Such PM macromolecular complexes regulate not only CFTR membrane levels but also its function [112].

Impact of mutations on CFTR membrane levels/stability was first reported for C-terminal truncations, as this region was found not be required for biogenesis and channel function but to be indispensable for maintaining the PM stability of mature CFTR [71].

If a mutation has an impact at these later stages of trafficking, rescue can be promoted using ‘stabilizers’ [113]. Although no such compounds are under pre-clinical development, strategies may include modulation of pathways that have been described to influence CFTR PM stability, including small GTPase Rac1 activation [114], cAMP sensor EPAC1 activation [115], inhibition of kinases such as SYK [116,117] or LMTK2 [118] or modulation of anchoring through PI3Kγ [119].

### Mutations affecting function

Mutations can also cause CF by affecting channel function – transcription, translation, processing, trafficking and membrane stability are unaffected but either gating or conductance is impaired.

As described above, opening and closing of the anion pore of CFTR is regulated by two main processes: first, the R domain needs to be phosphorylated, in general by cAMP-dependent protein kinase A (PKA); when phosphorylated, the R domain moves aside allowing the dimerization of the two NBDs driven by ATP binding. The NBDs form a head-to-tail dimer with two ATP binding sites located at the dimer interface. ATP binds tightly to one ATP-binding site (called site 1, which is formed by the Walker A and B motifs of NBD1 and the signature motif of NBD2) but is not hydrolyzed at this site 2. ATP also binds and is rapidly hydrolyzed at the other ATP-binding site (site 2, which is formed by the Walker A and B motifs of NBD2 and the signature motif of NBD1). Mutations that affect the ability of the NBDs to bind and hydrolyze ATP are likely to affect channel gating (and are grouped in class III).

Channel function is also dependent on the segments that line the anion pore and that contribute to anion selectivity. Mutations at such locations are likely to affect channel conductance (and are grouped in class IV).

3D structures, sometimes enriched by molecular dynamics (MD) simulations, have extensively been examined with the aim at understanding the possible impact of mutations. A lot of ‘functional’ mutations (class III (gating) and class IV (permeation)) form hot spots located in the pore and in the ATP-binding sites [31]. Class III mutations are in particular found at the level of the interface of the pseudo dimer formed by the NBDs (in particular in the ATP-binding sites) and by the ICLs [88]. Mutations are predicted to interfere with the tight association of the different subunit, which is required for the opening of the channel. The most common class III mutation, G551D, located within the canonical ATP-binding site, also impacts on phosphorylation-dependent activation of CFTR [120]. A strong gating defect is observed for the R117H mutation, located in the first extracellular loop ECL1 in MSD1. The effect of this mutation can be explained by a loss of a hydrogen bond formed with the carbonyl atom of a residue belonging to MSD2, which stabilizes the open form of the channel [121]. Gain-of-function (GOF) mutations, such as F337S or F337A [45,122], located at the level of the extracellular gate, K978C, within the inner vestibule [44,123], or D1341R and D173R, at the NBDs:MSDs interface [120], illuminate our global understanding of the gating mechanisms, relying on diverse mechanisms, from stabilization of the open state to the removal of inhibitory mechanisms. Study of GOF mutations has also emphasized the role of the modulation of ATP/PKA sensitivity in the activation of the channel [45,120].

The potential importance of TM8 discontinuity for gating was supported by the identification, always using cryo-EM, of a binding site for ivacaftor (VX-770), between TM8, TM4, TM5 and the lipid bilayer [124]. However, no conformational difference was observed between the structures solved in presence and absence of the drug, leaving unsolved the issue of the mechanism of potentiation. Molecular docking of chemotypes, followed by pharmacophore mapping and QSAR analysis gave useful information to further develop potentiators adapted to this site [110]. Other binding sites for ivacaftor, involving ICL4, have been proposed, based on hydrogen-deuterium exchange [125] and on the use of photoactivable probe analogs of VX-770 in biological membranes [126]. Several other potentiators have also been identified, some of which such as GLPG1836 sharing the same binding site than VX-770 [124,127], while other ones have complementary mechanisms of action and might be used as co-potentiators [128–133]. Potential binding sites at the NBD1:NBD2 interface have been proposed for the potentiators SBCs [128], while the binding sites of the other ones remain to be explored. A comprehensive mapping of potentiators binding sites will allow to understand the associated mechanisms of action and thereby identify key features of the gating mechanism. Of particular interest will be to understand the role of electrostatic interactions between charged residues and with lipids in the long-range conformational transitions and in the stabilization of the open form of the channel.

### Mutations affecting production and processing of mRNA

Among the many variants reported, some have an impact in the production of CFTR mRNA – affecting either the amount or length of mRNA produced. These include large insertions or deletions, splicing mutations, and nonsense mutations.

Depending on their location, splicing mutations can affect either the production of full-length mRNA or the amount produced. Splicing process is a complex multistep process during which the spliceosome, the major effector of the splicing reaction that includes many proteins and small nuclear RNAs (snRNAs) including the five small nuclear ribonucleoproteins (snRNPs) U1, U2, U4, U5 and U6, binds and recognizes specific sequences at the pre mRNA [134]. First, U1 binds to the 5′ splice site by complementarity and U2 binds to the branch point. The triple snRNPs (U4, U5 and U6) complex moves-in to associate with the assembling spliceosome. Then, the U4 leaves the complex, allowing the replacement of U1 by U6 that interacts with U2 to bring the branch point into proximity to the 5′-splice site. At this point, the first transesterification reaction cleaves the 5′-splice site of the intron of the downstream exon and attaches it to the branch point. U5 then brings the 3′-splice site of the upstream exon and 5′-splice site of the downstream exon into proximity with each other, allowing a second transesterification reaction that cleaves the 3′-splice site of the upstream exon. Splicing accuracy depends not only on the mechanisms described above but also on splicing regulatory elements which direct the splicing machinery to use the correct splice site [134].

Splicing mutations at the canonical splice sites have also major impact on the expression of CFTR, as they affect the sequences that define exon–intron boundaries. The 5′ splice site (sequence CAG/GUAAGU) and the 3′ splice site (sequence NYAG/G) are recognized by components of the spliceosome, and changes in these sequences affect interactions that regulate intron removal. Thus, mutations located at + 1 and + 2 residues at the 5′ donor splice site and at − 1 and − 2 residues at the 3′ acceptor splice site usually lead to single exon skipping.

From the 230 splicing alterations described in the CFTR Mutation Database, about 100 are at the canonical splice sequences at positions +/- 1 or 2 and thus likely to have major impact. As with large indels, these mutations also abolish the production of full length CFTR mRNA and therapeutic strategies directed at rescuing mRNA have limited hypothesis of success – thus, the focus should be to act either at the gene level or by circumventing CFTR.

The only exceptions occur if the splice site is weak and the presence of the mutation uncovers a cryptic splice site in a neighboring exon or intron that can be used in the splicing process. The use of alternative splice sites is very likely to occur for all the splicing mutations that do not occur at canonical sites. In those cases, instead of totally disrupting the recognition of an exon-intron boundary, mutations are likely to weaken the strength of the wild-type splicing process – leading to alternative splicing that many times occurs in parallel with the normal one. The cellular outcome of these mutations is a reduction in the levels of wt CFTR mRNA and protein (class V). This has been described for mutations such as 3849+10 kb C>T, 2789+5G>A, 3272-26A>G and several others [135–137].

In these cases, therapeutic strategies are designed to increase the occurrence of the normal splicing reaction by masking or preventing the alternative sites. Approaches to correct splicing mutations have been described using antisense oligonucleotides (AONs). These AONs are complementary to a particular RNA sequence and thus very specific. When designed to bind to splice sites, they can be used to modulate the splicing process in a precise and reproducible way. Using this approach, splice sites and regulatory can be blocked preventing the binding of the spliceosome components to the mutated site and favoring the splicing reaction at the normal site [138]. Although the RNA repair is transient and modestly effective, the levels of correction may be sufficient to improve or even eliminate disease phenotype [139].

Different studies have reported on the ability of AONs to correct splicing mutations in CFTR including 2789+5G>A and 3849+10 kb C>T [137,140]. Recent studies have in fact focused on the development of drug candidates for 3849+10 kb C>T using chemically modified AONs to serve as a basis for clinical evaluation of the identified lead molecules [141].

A second group of mutations that affect mRNA levels (or structure) are nonsense mutations. In this case, the presence of a premature termination codon (PTC) triggers a surveillance mechanism – called nonsense-mediated decay (NMD) – that recognizes and rapidly degrades the mRNA molecule, thus protecting the cell from the deleterious effects that could arise from the synthesis of a C-terminally truncated protein [142,143]. NMD depends on the interaction of the translation termination complex with a dynamic multiprotein assembly called exon junction complex (EJC). These complexes are essential to discriminate a PTC from a normal termination codon. EJCs are deposited 20–24 nts upstream the exon–exon junctions during splicing and remain associated with the mRNA during transport to the cytoplasm. During the pioneer round of translation, EJCs are displaced by the ribosome. When an mRNA contains a PTC located 50–54 nts upstream an exon–exon junction, the distal EJCs are not displaced triggering assembly of a degradation complex that controls NMD [144].

Approximately 8% of the variants described in the *CFTR* gene introduce a PTC and are thus likely to lead to severe CF phenotype. The position and/or the sequence context of a specific PTC have however an impact in the amount of mRNA that is degraded by NMD, as transcripts bearing PTC very close to the initiation codon or to the normal termination one having only a modest impact in transcript level [145].

Potential treatments for these mutations need to accomplish two major tasks: inhibit NMD and allow the readthrough of the PTC. To date, the only effective strategies to inhibit NMD include the use of SMG11i, a direct inhibitor of SMG1, one of the components of the degradation machinery – however, this molecule has significant cytotoxic effects and thus developments are needed to move it into clinical use [146].

Readthrough agents that promote insertion of near-cognate aminoacids in place of the PTC [147] include aminoglycoside antibiotics like gentamycin or tobramycin [148], ataluren/PTC-124 that was used in the context of CF but failed its endpoint in clinical trials [149] and more recently the investigational drug ELX-02 currently in clinical trials for patients bearing at least one copy of G542X, the most common nonsense mutation in the *CFTR* gene [150,151].

Finally, large insertions and deletions, which account for approximately 3% of the variants described in the *CFTR* gene, will lead to the production of mRNA that is significantly divergent from wt mRNA, either due to lack of substantial regions or to insertions that alter the open reading frame. Any corrective measure is likely to fail with such mutations, that can only be circumvented by strategies that do not target the endogenous *CFTR* gene or protein – and these may include gene replacement (as in gene therapy) or CFTR-bypass strategies (targeting different channels or transporters).

Gene therapy and gene editing offers great hope for the treatment of a monogenic disease such as CF as these approaches would mean a potentially permanent cure (reviewed in [152,153]). In the case of gene therapy, although preclinical studies evidenced some success in cell and animal models, results from clinical studies have evidenced several difficulties, namely targeting the correct cell population in a proportion enough to translate into clinical benefit or overcoming the mucus barrier, limiting the number of studies that have in fact aimed at developing gene therapy approaches for CF patients [154].

Strategies aimed at other channels and transporters (discussed and reviewed in [106]) are under development or clinical trial focusing on the inhibition of the epithelial sodium channel (ENaC), the modulation of the calcium activated channel TMEM16A (or anoctamin 1) or of the solute carrier family member 26A member 9 (SLC26A9). Some attention has also been given to the identification of synthetic anionophores, able to restore transmembrane chloride transport.

## Conclusion

**Figure 3:**
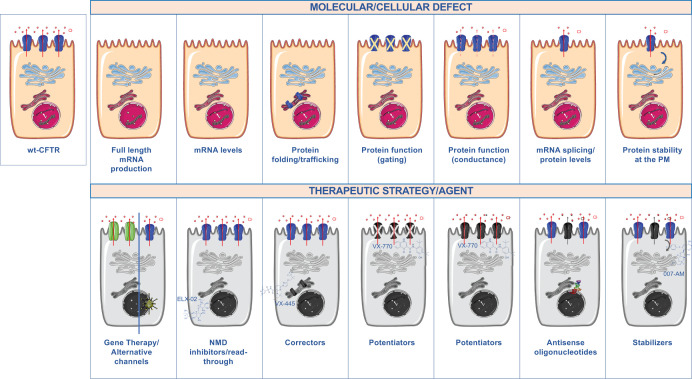
Mechanisms of disease for CFTR mutations Mutations can cause different molecular defects and that require specific rescuing strategies.

CF is a complex disorder, and this is mainly since, although all mutations impair or decrease conductance through the CFTR pore, the molecular mechanisms involved are quite diverse. Knowledge on these detailed mechanisms has informed the development of therapeutic strategies that target the fundamental molecular and cellular defects (summarized in Figure 3). Such advances have contributed significantly to make precision (and personalized) medicine a reality in the field.

## Abbreviations

AON: antisense oligonucleotide
BMI: body mass index
CF: cystic fibrosis
CFTR: cystic fibrosis transmembrane conductance regulator
EJC: exon junction complex
ENaC: epithelial sodium channel
GOF: gain-of-function
MD: molecular dynamics
MSD: membrane-spanning domain
NBD: nucleotide-binding domain
NMD: nonsense-mediated decay
PI: pancreatic insufficiency
PM: plasma membrane
PTC: premature termination codon
SLC26A9: solute carrier family member 26A member 9
